# Potential of the predatory mite, *Amblyseius swirskii* to suppress the Broad Mite, *Polyphagotarsonemus latus* on the Gboma Eggplant, *Solanum macrocarpon*

**DOI:** 10.1673/031.012.0701

**Published:** 2012-01-24

**Authors:** Alexis Onzo, Arnaud F. Houedokoho, Rachid Hanna

**Affiliations:** ^1^ international Institute of Tropical Agriculture, 08 B.P. 0932, Cotonou, Benin, West Africa; ^2^ international Institute of Tropical Agriculture, B.P. 2008 (Messa), Yaoundé, Cameroon; ^3^ Faculté d'Agronomie, Université de Parakou, B.P. 123, Parakou, Bénin, Afrique de l'Ouest

**Keywords:** African egg plant, Phytoseiidae, Tarsonemidae, biological control, solanaceous crops

## Abstract

In Benin, the tarsonemid mite *Polyphagotarsonemus latus* (Banks) (Prostigmata: Tarsonemidae) is a key pest of gboma eggplant *Solanum macrocarpon* (L.) (Solanales: Solanaceae), a leafy vegetable on which it causes considerable damage to the plants and substantial reduction in yield. Predatory mites in the family Phytoseiidae have been successfully used in the biological control of numerous agricultural pests worldwide. In that respect, a population of the phytoseiid mite *Amblyseius swirskii* (Athias-Henriot) (Mesostigmata: Phytoseiidae) has been identified as a potential predator of *P*. *latus*, and is now a candidate for release against this pest in Benin. The objective of the present study is to determine, through laboratory experiments, the predation rate and life table parameters of *A*. *swirskii* when feeding on *P*. *latus* or alternative food such as maize pollen. Under laboratory conditions the mean number of *P*. *latus* consumed by *A*. *swirskii*, and daily oviposition, significantly increased as the number of prey increased. Total development time of *A*. *swirskii* was significantly shorter when it fed on *P*. *latus* than on maize pollen. Net reproduction rate, intrinsic rate of increase, mean generation time and the finite rate of increase of *A*. *swirskii* were were all significantly lower on *P*. *latus* than on maize pollen. However, doubling time was significantly higher on maize pollen. This study shows that *A*. *swirskii* is a good predator of *P*. *latus*, and that maize pollen can efficiently sustain *A*. *swirskii* populations when *P*. *latus* densities on plants become low. Consequently, *A*. *swirskii* can be used for the biological control of the broad mite *P*. *latus* on gboma eggplant, and on other solanaceous crops in Benin and elsewhere.

## Introduction

In Benin, as in many other African countries, urban and periurban agriculture has recently attracted much interest and attention as a consequence of increasing food demand following increasing populations (AssogbaKomlan et al. 2007). Horticultural production, especially that of leafy vegetables, occupies an important part in the agricultural activities developed around main towns in southern Benin (Assogba-Komlan et al. 2007), where these vegetables are an important source of income (Oomen and Grubben 1978; Beniest 1987) from local sales and exports (Reckhaus 1997; Schippers 2000). Traditional leafy vegetables are very rich in many vitamins (especially A, B, and C), minerals, fibers, carbohydrates, and proteins, and several of them are known for their medicinal properties (Stevels 1990; Rehn and Espig 1991; Schippers 2000; Dansi et al. 2008). Therefore, they represent a low—priced nutritional food vital to households in rural as well as urban areas (Chweya and Eyzaguirre 1999).

The gboma eggplant, *Solanum macrocarpon* (L.) (Solanales: Solanaceae) is one of the most popular leafy vegetables in West and Central Africa (Oomen and Grubben 1978). It is the second most commonly grown and consumed traditional leafy vegetables in Southern Benin (Assogba-Komlan et al. 2007), behind *Amaranthus cruentus* L. (Hounkpodoté and Tossou 2001; Grubben 2004). Constraints to gboma eggplant production are mostly phytosanitary (Assogba-Komlan et al. 2007), among which is the broad mite, *Polyphagotarsonemus* latus (Banks) (Prostigmata: Tarsonemidae). Since its discovery in Southern Benin (Bordat and Goudegnon 1991), this polyphagous mite turns out to be the most dangerous pest of *S*. *macrocarpon* (Adango et al. 2006), and represents the key factor that constrains farmers from growing the gboma eggplant on most vegetable farms in urban and peri—urban zones throughout the country (James et al. 2007).

Once *P*. *latus* was recognized as a key pest of gboma, many chemical pesticides were tried to reduce its impact. However, the very small size of this mite pest and its preference for the lower surface of leaves (Jeppson et al. 1975; Gerson 1992) has hampered its control with the application of chemical pesticides. Moreover, leaves attacked by *P*. *latus* are deformed with curls, which make it difficult to reach the pest and complicate its control with non—systemic pesticides. These difficulties in controlling *P*. *latus* has prompted growers to apply inappropriate doses of chemicals often exceeding 2 to 5 times the recommended dosage, with associated impacts on the environment and on human and animal health (Assogba-Komlan et al. 2007). Although data are not available to show the presence of resistance to regularly applied pesticides, it is very likely that such resistance has developed as this polyphagous mite is known to quickly develop resistance to chemical pesticides (Peña 1999). The case of *P*. *latus* control on gboma eggplant has become a classic case of the pesticide treadmill that prompted the advancement of integrated pest management (Stern et al. 1959; Stern 1973). To overcome this obstacle the search for more durable crop protection solutions based on IPM systems on vegetable farms has become mandatory (Cranham and Helle 1985; Adango et al. 2007; James et al. 2007).

Biological control with natural enemies (predators, parasites, or pathogens) is a viable alternative to chemical control measures. Natural enemies can be native or introduced. The use of natural enemies prevents environmental risks associated with chemical pesticides, while sustainably protecting the crop, given that the biological control agent does not cause any harmful non—target effects.

Numerous predatory mite species in the family Phytoseiidae have been used for the biological control of phytophagous mites on cultivated crops (McMurtry and Croft 1997; Hanna et al. 2005). The predatory mite *Amblyseius swirskii* (Athias Henriot) (Mesostigmata: Phytoseiidae) had been identified as an effective predator against *P*. *latus* (Tal et al. 2007; van Maanen et al. 2010). This predatory mite species is presently candidate for introductive and augmentative releases against *P*. *latus* on vegetable farms in Benin. A prerequisite for the field introduction of *A*. *swirskii* against *P*. *latus* in Benin is the need for preliminary studies to quantitatively determine the capacity of the predator species to effectively feed and reproduce on the key pest (or prey) under the local growing conditions of the gboma eggplant in Benin; however, such data were lacking.

The primary objective of the present study is to determine the capacity of *A*. *swirskii* to suppress *P*. *latus* populations on *S*. *macrocarpon*. This was achieved through laboratory studies to determine the predation capacity of *A*. *swirskii* on *P*. *latus* through classical life table studies of *A*. *swirskii* on *P*. *latus* and on maize pollen as alternative food generally present within or around vegetable farms.

## Materials and Methods

### Study site

The experiments were conducted between May and November 2008 in the laboratory of Acarology at the Benin station of the International Institute of Tropical Agriculture (IITA-Benin) located at about 12 km Northwest of Cotonou. The geographical coordinates of this research station are 6° 25′ N, 2° 20′ E, with an elevation of 15 m above sea level.

### Plant materials

The *S*. *macrocarpon* variety used in these experiments was previously imported from Ghana while the seeds were supplied by the vegetables' research program of the National Institute of Agricultural Research in Benin (INRAB). For the production of *S*. *macrocarpon* plants, seeds of this crop were sown in a seedbed. Sprouted seedlings were transferred individually to plastic pots three weeks after planting. Potted plants were placed on nine iron benches, which were arranged at 65 cm apart inside a screenhouse. Plants were arranged in a spacing of 25 cm and watered regularly every other day. To ensure permanent availability of gboma plants for the study, some seedlings were also planted in a field at the IITA station on eight plots of 1.25 × 9 m with the spacing of 25 cm within and between lines. Field plots were fertilized using urea followed by chicken manure two weeks after transplanting the seedlings. Plants were watered every day early morning and late evening. No pesticides were applied in the neighborhood of the plots.

Pollen of maize *Zea mays* L. (Poales: Poaceae) and cattail, *Typha australis* (Cav.) Trin. ex Steudel (Typhaceae) were used as alternative foods for the rearing of *A*. *swirskii* in the laboratory (Nomikou 2003). Maize and typha pollens were regularly harvested from experimental plots setup at the station for this purpose. The pollens were collected in plastic tubes and preserved in a refrigerator at 10–15 ^°^C.

### Sources of mites

Individuals of *A*. *swirskii* used in the experiments were obtained from a colony maintained at IITA-Benin in a laboratory for 15 months at 27 ^°^C and 80% RH. This predatory mite was originally obtained from a laboratory colony from the University of Amsterdam where it has been maintained since its introduction from Israel in year 2000 (Nomikou 2003). Colonies of this predatory mite species were maintained on typha pollen and on two—spotted mite *Tetranychus urticae* Koch (Prostigmata: Tetranychidae) eggs.

Individuals of *P*. *latus* were collected in a *S*. *macrocarpon* plot installed on the experimental farm of ITA-Benin. They were then reared permanently throughout the experimental period either on *S*. *macrocarpon* maintained in the screenhouse or in new field plots on the IITA campus.

### Predation and prey conversion efficiency

For this study, a cohort of *A*. *swirskii* was initiated from one day—old eggs. After hatching, immature *A*. *swirskii* were fed with *P*. *latus* until they reached adult stage. Thirty adult females of the same age were then removed from the cohort and starved individually in a small plastic tube without any food for 24 hours. Subsequently, each predator was placed singly on *S*. *macrocarpon* leaf disc (2 cm diameter) placed abaxial surface up on water—saturated cotton wool in an open Petri dish (15 cm diameter). Each Petri dish held a maximum of 10 leaf discs that were at least 2 cm apart. Water was added onto the Petri dish to keep the leaf discs turgid while serving as barrier that prevented predators from escaping. Petri dishes were placed in a plastic (PVC) tray. The 30 predators were then separated into two batches of 15 adult *A*. *swirskii* females. Individuals of the first batch were fed daily with 25 adult females of *P*. *latus* while individuals of the second batch were fed daily with 50 adult females of *P*. *latus*. The number of added prey constituted the treatments while the 15 individuals of a batch represented the number of replicates.

The experiment was monitored daily at the same time period. During each monitoring period, the number of prey mites consumed and the number of eggs laid by each female *A*. *swirskii* were recorded; thereafter, the number of prey consumed was replaced from the stock colony as described above. Mites were transferred to leaf discs using a camel hairbrush. Food renewal was done under a binocular microscope due to the small size of the prey mite, *P*. *latus*. The leaf discs were replaced twice each week and the trial was conducted over a 15 day period.

### Development of immature stages of *A*. *swirskii*

This experiment was conducted in the laboratory in a Partlow MRC 7000 growth chamber (www.partlow.com) at 25 ± 1 ^°^C, 80 ± 10% RH and a photoperiod of 12:12 L:D.

Several gravid females *A*. *swirskii* were grouped on an arena where they were allowed to lay eggs. A total of 120 eggs deposited within a period of 24 hours were collected and placed individually on *S*. *macrocarpon* leaf discs (approximately 2.5 cm diameter). Leaf discs were kept in a Petri dish (15 cm diameter) on water soaked cotton wool. Each Petri dish contained 10 leaf discs. Water was regularly added in the Petri dish to keep the leaf discs turgid while constituting a barrier that prevent predators from escaping. Each Petri dish constituted a replicate, and a total of 8 Petri dishes were distributed equally among two treatments: (T1) *P*. *latus* (all stages combined) provided *ad libitum*, and (T2) maize pollen provided *ad libitum*. Source and handling of *P*. *latus* and maize pollen are described above.

Leaf discs were replaced twice a week for the treatment T1 (i.e., *P*. *latus*) because of the mite feeding damage, and once a week for the treatment T2 (maize pollen). Each treatment was repeated 40 times. The developmental stage reached by each individual was recorded until it reached adult stage. The presence of exuvia was used as evidence of molting. Replicates in which predators escaped from leaf discs were eliminated from further consideration. The experiment was monitored each day at 07:00 and 19:00 when the amount of prey and pollen was renewed or supplemented to prevent predators from starving. The durations of each immature stages (egg, larva, protonymph, and deutonymph) were recorded per treatment.

### Population growth studies

The study was conducted both on *P*. *latus* and on maize pollen. The life table characteristics of the predator were determined separately on two groups of the predators maintained on the two food types: *P*. *latus* or maize pollen, representing the two treatments. Both *P*. *latus* and maize pollen were provided *ad libitum*.

The experiments were conducted in the growth chamber at 25 ± 1°C, 80 ± 10% RH and 12:12 L:D. Gravid *A*. *swirskii* females were grouped on an arena to initiate a cohort of 200 eggs laid within a 24—hour period. For each treatment, 100 eggs were used in order to have sufficient number of female *A*. *swirskii*. Each egg was isolated on a leaf disc cut from leaves of gboma and placed in a Petri dish (15 cm diameter). Each Petri dish contained 10 leaf discs; five Petri dishes were arranged in a plastic PVC tray.

Immediately after the deutonymphal stage was reached, the sex of the predators was determined. To ensure mating, two adult males obtained from the mother colony were placed with each newly emerged adult female on the leaf disc. Males were removed from the arenas once egg—laying had started. Ovipositing females were transferred to new leaf discs twice a week in the *P*. *latus* treatment and weekly in the maize pollen treatment. The number of eggs deposited by each female was recorded daily until all females died. Eggs obtained from each female were grouped per treatments to determine their hatchability, survival rates of the immature stages, and sex ratio of the adults. During this stage of the experiment, all groups obtained from ovipositing females were maintained on *T*. *australis* pollen as food.

The following life history parameters were estimated: pre—oviposition, oviposition, and post-oviposition periods; female fecundity (i.e., the average number of eggs laid by female per day), the longevity of the female (i.e., the average life span of a female from egg stage until its death). Population growth parameters calculated included the intrinsic rate of natural increase (*r_m_*), the net reproductive rate (*R_o_*); the mean generation time (*T_g_*); the population doubling time (*T_d_*); and the finite rate of increase (λ). These are the parameters most commonly used to describe arthropod populations (Maia et al. 2000; Riis et al. 2005). These parameters were estimated using the method described by Andrewartha and Birch (1954) and calculated using the Jacknife program developed by Maia et al. (2000) in SAS 9.1 (version 2003). In addition, the sex—ratio and the survival rates of juvenile and adult stages were calculated.

#### Statistical analyses

The mean number of *P*. *latus* consumed each day and the mean number of eggs laid daily by each predator were estimated for each treatment (prey density). The effect of prey density on daily prey (i.e., number of *P*. *latus*) consumption and number of eggs laid by the predatory mite were determined using Proc *t*— test in SAS (2003). Similarly, duration of each developmental stage of the predator was compared between the two treatments using Proc *t*—*test* in SAS (2003). Data were log-transformed (i.e., log_10_(x +1)) before their use in the statistical analyses. As for the different growth parameters, they were compared between the two treatments using an analysis of variance (Proc ANOVA) in SAS (2003).

**Figure 1.**
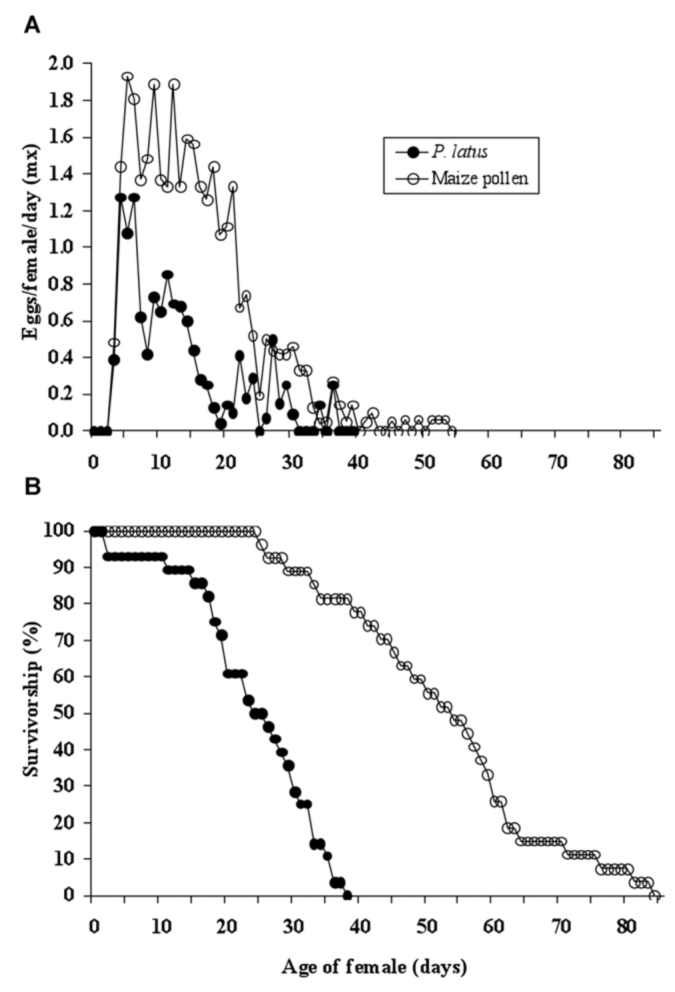
(a) Fecundity and (b) age—specific survival of *Amblyseius swirskii* on two food sources (*Polyphagotarsonemus latus* and maize pollen). Day 0 is the day on which the females became adults. High quality figures are available online.

**Table 1.**
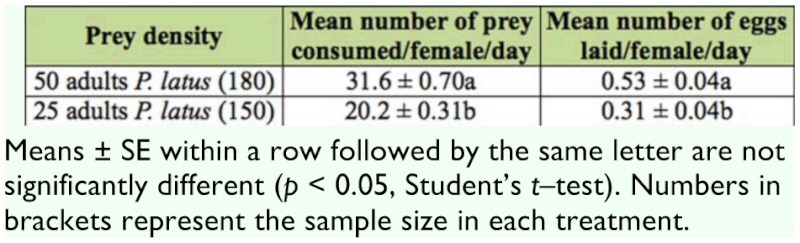
Predation and oviposition rates of *Amblyseius swirskii* (mean ± SE) on two densities of the broad mite *Polyphagotarsonemus latus* at 25 ± 1^°^C and 80 ± 10% RH.

**Table 2.**
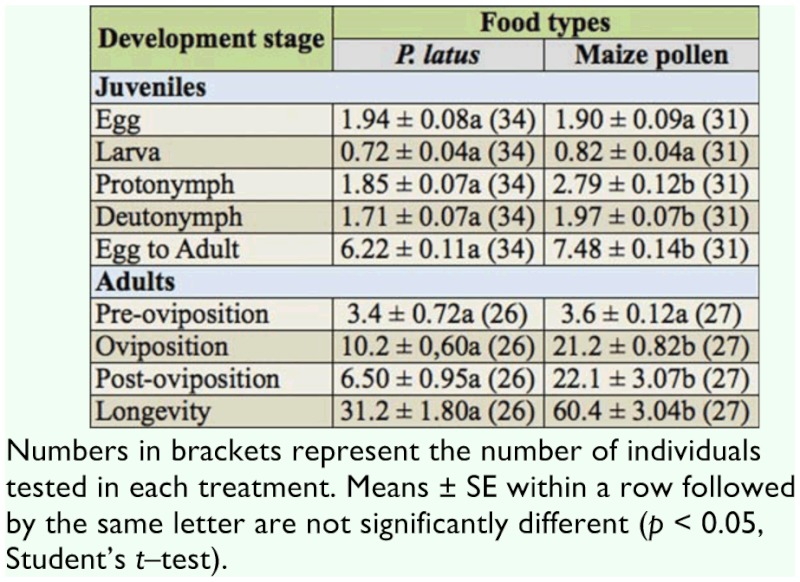
Mean development duration (± SE) of juvenile stages and adult females of *Amblyseius swirskii* on *Polyphagotarsonemus latus* and maize pollen at 25 ± 1^°^C and 80 ± 10% RH.

## Results

### Effect of *P*. *latus* density on predation rates on fecundity of *A*. *swirskii*

The average number of *P*. *latus* consumed per female *A*. *swirskii* per day increased significantly when the number of prey was increased from 25 and 50 adult females (Table 1; df = 328, *t* = -14.3, *p* < 0.01). Average daily oviposition per female *A*. *swirskii* also increased significantly when the number of prey was doubled (df = 328, *t* = -14.3, *p* < 0.01).

**Table 3.**
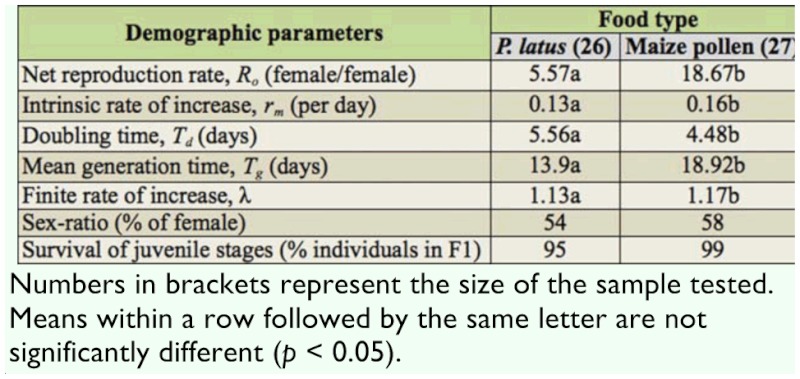
Demographic parameters of *Amblyseius swirskii* fed with *Polyphagotarsonemus latus* or maize pollen.

### Effect of food types on the duration of the developmental stages of *A*. *swirskii*

Duration of the egg and the larval stages were similar when *A*. *swirskii* was fed on *P*. *latus* or maize pollen (df = 63, *t* = 0.31, *p* = 0.76 for egg; df = 63, *t* = -1.66, *p* = 0.10 for larva) (Table 2). In contrast, durations of the protonymphal and deutonymphal stages of *A*. *swirskii* were significantly shorter when fed with *P*. *latus* than when fed with maize pollen (df = 63, *t* = -7.03, *p* < 0.01 for protonymph; and df = 63, *t* = -2.64, *p* < 0.05 for deutonymph).

Total developmental duration from egg to adult was significantly shorter on *P*. *latus* than on maize pollen (df = 63, *t* = -7.27, *p* < 0.01) (Table 2). However, the pre—oviposition period was not significantly different between the two food types (df = 51, *t* = -0.34, *p* = 0.73). Oviposition and post—oviposition periods were significantly shorter on *P*. *latus* than on maize pollen (df = 51, *t* = -10.70, *p* < 0.01 for oviposition period; df = 51, *t* = -4.79, *p* < 0.01 for post—oviposition period). Longevity of *A*. *swirskii* was significantly shorter on *P*. *latus* than on maize pollen (df = 51, *t* = -8.06,*p* < 0.01).

### Fecundity and survival of *A*. *swirskii *on *P*. *latus *and maize pollen

After adult emergence, egg production started on the same day for predators fed with *P*. *latus* and those fed with maize pollen (Figure 1a). Daily egg production of *A*. *swirskii* was significantly higher on maize pollen than on *P*. *latus* (df = 51, *t* = -12.21, *p* < 0.01). Egg production peaked on the 5^th^ day for *A*. *swirskii* fed on *P*. *latus* and the 6^th^ day for those fed on maize pollen (Figure 1a).

Survival of adult female *A*. *swirskii* (Figure 1b) decreased more rapidly on *P*. *latus* than on maize pollen. On the 24^th^ day, 50% of *A*. *swirskii* fed with *P*. *latus* had died, whereas almost all the predator fed with maize pollen were still alive. On the 38^th^ day all the predators fed with *P*. *latus* had died, whereas on that day more than 80% of predators fed with maize pollen were still alive.

### Population growth statistics of *A*. *swirskii *on *P*. *latus *and maize pollen

Life table parameters are presented in Table 3. Parameters such as the intrinsic rate of natural increase (*r_m_*), the net reproductive rate (*R_0_*), the mean generation time (*T_g_*), and the finite rate of increase (λ) were significantly lower for *A*. *swirskii* fed *P*. *latus* than maize pollen. Similarly, the proportion of female progeny and juvenile survival were also lower on *P*. *latus* than on maize pollen. In contrast, doubling time (*Td*) was longer on *P*. *latus* than on maize pollen.

## Discussion

Many studies were conducted on the search of biocontrol agents that are able to reduce or suppress the populations of *P*. *latus* on crop plants. However, very few of them focused on the potential of the predatory mite *A*. *swirskii* to control populations of *P*. *latus*, especially on gboma eggplant. Our study is among the few studies that show that *A*. *swirskii* could feed and reproduce effectively on *P*. *latus* as prey (Tal et al. 2007; van Maanen et al. 2010). Our predation test showed that a female *A*. *swirskii* could consume on average up to 32 female *P*. *latus* per day. The predator also showed a very positive functional response as the number of prey consumed increased with the amount of prey available. It was also shown that the predator could respond numerically by increasing its reproduction with increasing prey densities. Together, the positive functional and numerical responses to increasing prey densities provide strong support to the high capacity of *A*. *swirskii* to suppress *P*. *latus* populations.

The ability of *A*. *swirskii* to develop on a very widely available alternative food is another major positive aspect for the predator as biocontrol agent. Indeed, the voracity of *A*. *swirskii* could affect its survival when it reduces *P*. *latus* populations. However, our results showed that the predatory mite is able to survive, grow, and reproduce on a diet made exclusively of maize pollen, even though it develops faster on *P*. *latus*. The length of the nymphal stages were significantly shorter for *A*. *swirskii* fed on *P*. *latus* as compared to maize pollen, but this did not occur for the egg and larval stages. This was expected as the larval stages do not consume as much as the nymphal stages, therefore, their durations would not be expected to be affected by food type. In contrast, oviposition period and total lifespan of *A*. *swirskii* were two times longer on maize pollen than on *P*. *latus*. Similarly, the number of eggs laid per day is higher on maize pollen than on *P*. *latus*. This shows that maize pollen is a suitable food for the survival, development, and reproduction of *A*. *swirskii*.

Our results are similar to those of McMurtry et al. (1984) who observed that, when fed with pollen, the fecundity of predators was considerably higher than when offered *P*. *latus*. This is probably because the nutritional composition of pollen is more favorable for egg production than *P*. *latus*. For rearing A *swirskii*, the higher predation cost associated with feeding on a living prey compared with feeding on pollen would make the conversion of food to eggs to be more efficient with pollen than with *P*. *latus*.

In our study, the daily fecundity of *A*. *swirskii* on *P*. *latus* (1.15 ± 0.02 eggs/female/day) was lower than the 1.7 ± 0.13 eggs/female/day found by Momen and Abdel-Khaled (2008), when *A*. *swirskii* was fed with the eriophyid mite *Aculops lycopersici*, but similar to that found by El-Sherif et al. (1999) when *A*. *swirskii* was fed with *Tyrophagous putrescentiae*. Our value is higher than those obtained by Ragusa and Swirskii (1977) when *A*. *swirskii* was fed with Coccidae and Pseudococcidae (0 egg/female/day) or with *Tetranychus cinnabarinus*. Abou-Awad and Elsawi (1992) found that, when *A*. *swirskii* was maintained at 27 ^°^C over several generations on a diet of the two—spotted mite *Tetranychus urticae*, female predators laid between 1.11 and 1.45 eggs/female/day, which is comparable to our results.

The demographic parameters show that whether nourished with *P*. *latus* or with maize pollen, all juvenile stages of *A*. *swirskii* developed to adulthood. This is further evidence of the adequacy of the food offered to *A*. *swirskii* during our study, because when the food is not adequate, the survival rates of juvenile stages of *A*. *swirskii* are generally very low or zero (Ragusa and Swirskii 1977). Values of the various demographic parameters of *A*. *swirskii* were in general significantly different between the two food sources. With the exception of doubling time (*Td*), which was longer on *P*. *latus* than on maize pollen, values of the other parameters were lower on *P*. *latus* than on maize pollen. On *P*. *latus*, the population of *A*. *swirskii* increased 0.13 times per day against 0.16 times on maize pollen. Taken together, population increase rates and doubling time (5.56 on *P*. *latus* and 4.48 on maize pollen) show that *A*. *swirskii* populations can grow faster on maize pollen than on *P*. *latus*.

However, our results show that mean generation time was much shorter on *P*. *latus* (13.78 days) than on maize pollen (18.92 days), suggesting the adaptation of *A*. *swirskii* to *P*. *latus* as prey. Therefore, *A*. *swirskii* would make a very good predator of the broad mite *P*. *latus* on gboma eggplant. The good performance of *A*. *swirskii* on maize pollen suggests that it could be reared in mass on pollen as an effective biocontrol agent against mites. Maize pollen can be found in high quantities in or around vegetable farms during much of the year, and this alternative food could naturally support populations of *A*. *swirskii* when *P*. *latus* become scarce, thereby sustaining the predator on vegetable farms. The major constraint for the use of *A*. *swirskii* as a biocontrol agent on gboma farm is to instruct and convince vegetable growers to refrain from applying chemical pesticides on or around the vegetable farms, since phytoseiids predators are very sensitive to chemical pesticides (Adango et al. 2007).
